# Nicotine and Cardiovascular Health: When Poison is Addictive – a WHF Policy Brief

**DOI:** 10.5334/gh.1292

**Published:** 2024-01-31

**Authors:** E. Ulysses Dorotheo, Monika Arora, Amitava Banerjee, Eduardo Bianco, Nuan Ping Cheah, Regina Dalmau, Thomas Eissenberg, Koji Hasegawa, Pamela Naidoo, Noreen T. Nazir, L. Kristin Newby, Nour Obeidat, Andrii Skipalskyi, Janina Stępińska, Jeffrey Willett, Yunshu Wang

**Affiliations:** 1 Southeast Asia Tobacco Control Alliance, Philippines; 2 Public Health Foundation of India, India; 3 University College London, United Kingdom; 4 Amrita Institute of Medical Sciences, India; 5 Frank Foundation for International Health, Uruguay; 6 Health Sciences Authority, Singapore; 7 University Hospital la Paz, Madrid, Spain; 8 Virginia Commonwealth University, United States; 9 National Hospital Organization Kyoto Medical Center, Japan; 10 Heart and Stroke Foundation South Africa, South Africa; 11 University of the Western Cape, South Africa; 12 University of Illinois Chicago, United States; 13 Duke University School of Medicine, United States; 14 King Hussein Cancer Center, Jordan; 15 World Health Organization, Ukraine; 16 Department of Medical Communication, School of Public Health, Centre of Postgraduate Medical Education, Warsaw, Poland; 17 American Heart Association, United States; 18 World Heart Federation, Switzerland

**Keywords:** Tobacco and Nicotine Products, Risk Factor for Cardiovascular Disease, Tobacco Control and Public Health

## Abstract

Nicotine is universally recognized as the primary addictive substance fuelling the continued use of tobacco products, which are responsible for over 8 million deaths annually. In recent years, the popularity of newer recreational nicotine products has surged drastically in many countries, raising health and safety concerns. For decades, the tobacco industry has promoted the myth that nicotine is as harmless as caffeine. Nonetheless, evidence shows that nicotine is far from innocuous, even on its own. In fact, numerous studies have demonstrated that nicotine can harm multiple organs, including the respiratory and cardiovascular systems.

Tobacco and recreational nicotine products are commercialized in various types and forms, delivering varying levels of nicotine along with other toxic compounds. These products deliver nicotine in profiles that can initiate and perpetuate addiction, especially in young populations. Notably, some electronic nicotine delivery systems (ENDS) and heated tobacco products (HTP) can deliver concentrations of nicotine that are comparable to those of traditional cigarettes. Despite being regularly advertised as such, ENDS and HTP have demonstrated limited effectiveness as tobacco cessation aids in real-world settings. Furthermore, ENDS have also been associated with an increased risk of cardiovascular disease.

In contrast, nicotine replacement therapies (NRT) are proven to be safe and effective medications for tobacco cessation. NRTs are designed to release nicotine in a slow and controlled manner, thereby minimizing the potential for abuse. Moreover, the long-term safety of NRTs has been extensively studied and documented.

The vast majority of tobacco and nicotine products available in the market currently contain nicotine derived from tobacco leaves. However, advancements in the chemical synthesis of nicotine have introduced an economically viable alternative source. The tobacco industry has been exploiting synthetic nicotine to circumvent existing tobacco control laws and regulations. The emergence of newer tobacco and recreational nicotine products, along with synthetic nicotine, pose a tangible threat to established tobacco control policies. Nicotine regulations need to be responsive to address these evolving challenges. As such, governments should regulate all tobacco and non-medical nicotine products through a global, comprehensive, and consistent approach in order to safeguard tobacco control progress in past decades.

## Introduction and Significance

Cardiovascular disease remains the leading cause of mortality worldwide, claiming over 20.5 million lives in 2021 [1]. Tobacco use is recognized universally as a major risk factor for cardiovascular morbidity and mortality, contributing to approximately 17% of all deaths from cardiovascular disease [2,3]. Nicotine is widely acknowledged as the principal addictive substance driving the continued use of tobacco products, which are responsible for over 8 million deaths globally each year, including 1.68 million from ischemic heart disease [4,5].

Commercial tobacco and recreational nicotine product manufacturers, as well as proponents of their products, claim that nicotine is as harmless as caffeine [6,7,8]. They generally argue that the health harms from tobacco smoking, including its effect on cardiovascular disease and cancers, are attributable to the smoke and tar generated by the combustion of tobacco, rather than nicotine. Citing [medical] nicotine replacement therapy (NRT), which is non-combusted, as being widely accepted as safe [9,10,11], they frequently quote Dr. Michael Russell, who wrote in 1976, ‘*People smoke for nicotine, but they die from the tar*’ [12]. Many manufacturers aggressively market non-combusted forms of recreational nicotine delivery, such as electronic nicotine delivery systems (ENDS) and heated tobacco products (HTP), as significantly less harmful than conventional cigarettes [13]. However, scientific evidence describing the adverse effects of nicotine on respiratory and cardiovascular health, cancers, brain maturation, and smoking cessation raises ongoing concerns about use of these nicotine delivery products [14,15], which also deliver other harmful substances in addition to potentially addictive levels of nicotine.

The tobacco industry’s history of promotional tactics to perpetuate nicotine dependence includes misleading claims about the health benefit of cigarette filters and ‘light’ or ‘low-tar’ cigarettes [16,17,18,19,20] and public denial of the addictive nature of nicotine, while manipulating the content of tobacco cigarettes to enhance their addictiveness [21,22,23]. Despite robust evidence, some proponents of ENDS, also commonly referred to as e-cigarettes, continue to question the addictive potential of nicotine, sometimes invoking the fact that nicotine is naturally present, in infinitesimal concentrations, in several edible plants of the nightshade family, and pointing out that there are no cases of addiction or health harms associated with the consumption of tomatoes, eggplants, and potatoes [24,25]. These claims and arguments are used systematically by the tobacco industry to lobby for minimal governmental regulation of products it labels as ‘alternative’ and ‘reduced risk.’

In light of continued industry misinformation about nicotine, further heightened by the commercial interest generated by a global proliferation of its newer delivery systems, the present document reviews the latest scientific evidence on nicotine and cardiovascular health in order to raise awareness of the potential harmful effects of nicotine on the cardiovascular system and provide evidence-based recommendations to the heart health community, policy makers, and the public at large.

## Sources and Chemistry of Nicotine

Nicotine is a colourless, odourless, oily liquid that turns brown when exposed to light or air [26]. It is the primary alkaloid of tobacco, as well as the most abundant pyridine alkaloid found in the leaves of plants from the Nicotiana genus [27]. *N. tabacum L*. and *N. rustica L*. are the major species used in the manufacturing of tobacco products due to the high levels of nicotine present in these species [28,29]. Typical nicotine concentrations range from 15 to 35 mg per gram (g) of tobacco, with total alkaloid concentrations reaching up to 79 mg per g of tobacco [30,31].

Nicotine belongs to a class of chemical compounds that displays *chirality*, meaning that the molecule exists in two forms that are non-superimposable mirror images of each other [32]. The most common nomenclature used to differentiate two chiral forms, also known as enantiomers, is the (R)- and (S)- system. Enantiomers possess the same physical and chemical properties but may considerably differ in their biological and pharmacological effects – e.g., one may be active, while the other may be inactive or even toxic [33,34]. As such, the (R)- and (S)- configurations of nicotine are classified as distinct chemical substances with unique Chemical Abstracts Service (CAS) Registry Numbers [35]. Nicotine extracted from tobacco plants consists predominantly of the (S)-form, which represents approximately 90% of total alkaloids present in tobacco, with the (R)-form only accounting for 0.1 to 4.32% of total alkaloids [36,37,38]. The toxicological, pharmacological, and biological profiles of (S)-nicotine have been studied extensively [39,40]. However, data and studies on (R)-nicotine and its effect on human health currently are limited.

The chemical structure of nicotine comprises a pyrrolidine ring connected to a pyridine ring. The two nitrogen centres allow nicotine to exist in three different protonated states, depending on its interactions with acids that are naturally present in tobacco leaves. These forms are namely free-base nicotine (Nic), mono-protonated nicotine (NicH^+^), and di-protonated nicotine (NicH_2_^2+^) [41]. Protonated forms, also known as nicotine salts, predominate in unprocessed tobacco leaves, while different ratios of free-base and salts exist in tobacco and recreational nicotine products. The significance of these forms lies in their ability to alter the addiction potential of nicotine [41].

### Synthetic nicotine

Increased commercial interests have driven up the demand for synthetic nicotine, as manufacturers exploit loopholes in tobacco control laws by shifting to chemically synthesized ‘tobacco-free nicotine.’ In addition, demand is driven by misleading tobacco industry promotion of ‘tobacco-free’ nicotine as cleaner, purer, and safer than tobacco-derived nicotine [42,43,44].

Nicotine extraction from tobacco plants remains the most economically efficient source. Historically, due to its chiral nature, production of nicotine through chemical synthesis has been very costly. However, synthetic nicotine is set to become profitable as industry efforts to refine existing techniques have been successful in developing economically viable chemical synthesis processes to produce synthetic nicotine [45]. In fact, several published patents describing commercially applied processes to synthesize nicotine have been adopted to produce nicotine in bulk industrial scale settings [46,47,48,49]. As a result, the difference of production costs between natural and synthetic nicotine has narrowed over the years, and synthetic nicotine is expected to become cheaper over time.

Synthetic nicotine is synthesized chemically to reproduce the effects of tobacco-derived nicotine. Three different compositions can be synthesized depending on the process: (i) pure (S)-form, (ii) a mix of both (S)- and (R)-nicotine forms, or (iii) pure (R)-form (Table 1). Currently, the composition of many commercialized synthetic nicotine products appears to contain a mixture of both nicotine forms [42,50].

**Table 1:** Chemical information for nicotine in different forms.


COMMON CHEMICAL NAMES	UNIQUE IDENTIFIER: CAS REGISTRY NUMBERS*	ORIGIN

(S)-nicotine / (S)-(-)- nicotine / (-)-nicotine	54-11-5	Tobacco plant (S > 90%; R < 5%) Chemically synthesized

(R, S)-nicotine mixture / (±)-nicotine	22083-74-5	

(R)-nicotine / (R)-(+)-nicotine / (+)-nicotine	25162-00-9	


* A Chemical Abstracts Service Registry Number is a unique numerical identifier assigned to a specific chemical substance [35].

### Nicotine pharmacokinetics

Due to its physical and chemical properties, nicotine can be absorbed via sublingual, buccal, intranasal, inhalational, and transdermal routes [36]. Tobacco and recreational nicotine products, as well as NRTs, are available in numerous types and forms that deliver varied levels of nicotine.

Nicotine in conventional cigarette smoke is inhaled primarily via fine and ultrafine particles, followed by a rapid diffusion of nicotine into the vapor phase within the lungs, allowing nicotine to reach the brain within 10 seconds. Each puff of a cigarette delivers about 100 to 150 ug of nicotine, and a cigarette generally delivers 1 to 2 mg of nicotine to the systemic circulation [51]. Studies show that cigarettes achieve higher and faster nicotine peaks than cigars, pipes, and most other forms of nicotine delivery [36,51].

Other smoked tobacco products, such as waterpipes, cigars and bidis, deliver variable quantities of nicotine that may be comparable to or higher than those delivered by a conventional cigarette (time to peak concentrations are variable) [52]. More recently, HTPs have emerged that deliver up to 83% of the nicotine observed with conventional cigarettes [53]. ENDS do not contain tobacco, but the aerosol they generate, particularly from newer generation devices, has been shown to deliver nicotine levels comparable to the conventional cigarette (albeit with longer time to peak concentration) [54].

The bioavailability of nicotine from smokeless tobacco products varies by product but tends to be lower than that of inhaled tobacco products [36].

Conversely, nicotine levels delivered by NRTs through transdermal, oral, or intranasal routes generally are much lower than levels observed with tobacco products and furthermore demonstrate more consistent delivery profiles [55].

Nicotine is metabolized into more than 20 metabolites in the liver, with cotinine and *trans* 3’-hydroxycotinine as the principal metabolites [36]. The latter are typically used as biomarkers to assess exposure to tobacco or nicotine [36,56,57].

The bioavailability of ingested nicotine is generally reduced due to first-pass metabolism in the liver [36]. As such, the infinitesimal quantities of nicotine present in tomatoes, eggplants, and potatoes do not reach the systemic circulation to exert any physiologic effects [25].

## Nicotine-Containing Consumer Products

A wide range of commercial tobacco and recreational nicotine-containing products is available globally, which has expanded in recent years with the introduction of products containing synthetic nicotine [43]. They differ in their formulation, method of nicotine delivery, pharmacokinetic profile of nicotine delivered, source of nicotine used (tobacco-derived or synthetic), toxicant content and emissions, health risks associated with their use, and how they are regulated. Products that contain and burn tobacco (e.g., cigarettes and shisha) pose well-known health risks, including cardiovascular disease and cancer, due to the hundreds of toxic chemicals and carcinogens in tobacco smoke. Smokeless tobacco products (e.g., chewing tobacco and snuff) also contain similar toxicants and pose similarly well-known health risks [58,59,60,61,62]. Although ENDS and HTPs are shown to emit lower concentrations of some toxicants in cigarette smoke (hence characterized by the tobacco industry as ‘reduced risk’ or ‘modified risk’ tobacco or nicotine products) [63,64], there has been growing evidence of increased risk of cardiovascular and other health harms from ENDS and HTP use [65,66,67,68]. Also, with ENDS and HTPs being fairly recent, no studies exist to support claims of positive health outcomes associated with their long-term use (i.e., continued, daily use for 20–30 years).

As of 2020, the global prevalence of current tobacco use among adults (aged 15 years and older) is 22.3%. The prevalence of current tobacco smoking is 17% among adults, 91% of whom are cigarette smokers. The prevalence of current smokeless tobacco use among adults is 6% [69]. The prevalence of current adult ENDS use varies widely by country, with the highest reported estimates between 8% and 11% [43,70]. Given their relatively recent introduction into the global market, the prevalence of HTP use is lower, approximately 1.3% in countries where HTP use has been evaluated [71]. Importantly, while decades of research have demonstrated the dependence, disability, disease, and death caused by commercial tobacco use, newer products such as ENDS, HTP, and oral nicotine pouches have been studied less; thus, any adverse health consequences of their long-term use are uncertain due to the lack of reliable data available to support relative risk assessments on disease outcomes [72].

### Combustible tobacco products

#### Cigarettes, bidis, and kreteks

The conventional cigarette is the most used commercial tobacco product globally. While the basic premise of combusted tobacco leaf wrapped in paper has remained consistent for the past century or more, over time, industry modifications have included adding plastic filters, sweet and minty flavourings, and coolants such as menthol [73]. Bidis are small hand-rolled cigarettes manufactured primarily in India and other Southeast Asian countries. Kreteks are cigarettes containing cloves and other flavourings, made and sold primarily in Indonesia [74].

#### Cigars

The cigar category includes filtered cigars, little cigars, cigarillos, cheroots, and large/traditional cigars, which include ‘premium’ cigars [75]. In some markets, filtered cigars containing less than 1.36 g of tobacco and close to the same size as cigarettes are categorized as little cigars. Cigarillos contain roughly 3 g of tobacco and are slightly larger than a traditional cigarette.

#### Pipe tobacco

Pipe smoking is the oldest form of tobacco smoking. Pipes come in various shapes and sizes. A pipe used largely in Arab countries is the midwakh, which burns a blend of tobacco leaves and barks and herbs called the dokha, which is known to contain and deliver high concentrations of nicotine within a few puffs [76].

#### Waterpipe (shisha/hookah) tobacco

Waterpipe tobacco involves the combustion or heating of tobacco mixtures (maassel) using ignited charcoal. Waterpipe tobacco smoke contains many of the same toxicants found in cigarette smoke, as well as toxicants produced by the burning charcoal, and, similar to other tobacco products, waterpipe tobacco is sold in a wide range of flavours and includes menthol varieties [77].

### Electronic nicotine delivery systems (ENDS)

Electronic nicotine delivery systems (ENDS), more commonly known as electronic cigarettes or e-cigarettes, refer to a wide range of battery-powered devices that heat a nicotine-containing liquid, gel, or solid to create an aerosol to be inhaled by the user [78]; some devices are capable of delivering nicotine concentrations comparable to or greater than those in traditional cigarettes [54]. These devices include refillable tank systems, pod systems that use cartridges, and single-use disposable systems. Since their emergence, ENDS have been promoted and studied as smoking cessation tools. Some randomized clinical trials have shown efficacy in smoking cessation in structured settings over relatively short-term use; however, reviews of evidence that include longitudinal studies of real-world users do not support their efficacy for cessation [79,80,81], and a large proportion of ENDS users (often a majority) continue to smoke (dual use) [82,83]. To date, no ENDS manufacturer has officially registered its products as smoking cessation devices with relevant national agencies. ENDS liquids are sold with variable concentrations of nicotine and in a wide range of flavours, including many youth-appealing flavours such as fruit, candy, mint, and menthol [84].

### Heated tobacco products (HTP)

Heated tobacco products (HTP) are battery-powered devices that create an aerosol by heating a stick of specially prepared tobacco, sometimes in combination with a liquid or gel [85,86]. The paper-wrapped tobacco sticks come in a range of flavours including menthol, mint, and other cooling flavours and are also promoted for smoking cessation, despite never having been registered for such use and evidence of their lack of efficacy for cessation [87].

### Smokeless tobacco and nicotine products

Smokeless tobacco, usually chopped and moistened, is used by chewing or holding it in the mouth between the gum and the cheek. Chewing tobacco is often mixed with herbs, spices, areca nut, betel leaf, or slaked lime. The global oral tobacco product (OTP) market has diversified greatly in recent years. In addition to moist/dry snuff products, including snus, the tobacco industry has introduced new recreational nicotine-delivering OTPs, often flavoured, including pouches, tablets, lozenges, gums and gummies, and toothpicks [88,89,90].

## Physiological and Clinical Effects of Nicotine on the Cardiovascular System

Nicotine is a major component in all tobacco products and plays a vital role in their harmful cardiovascular effects. Nicotine exerts its effects via stimulation of the nicotinic acetylcholine receptors (nAChRs) located in the central nervous system, at inter-ganglionic junctions of the autonomic nervous system, and on target organs throughout the body as part of the parasympathetic autonomic nervous system [73]. As a result of the global expression of these receptors, their stimulation causes broad physiologic effects, such as free radical production, inflammation, vascular wall adhesion and atherosclerosis (Table 2) [91,92,93,94,95,96].

**Table 2:** Effects of nicotine on cardiovascular parameters and risk factors.


PARAMETERS	EFFECTS OF NICOTINE	POTENTIAL COMPLICATIONS

Heart rate	Increase	Acute tachycardia

Blood pressure	Increase	Acute hypertension

Coronary vasodilator reserve	Decrease	Myocardial ischemia (chest pain)

Action on blood vessels		Hypertension

Cutaneous vessels	Vasoconstriction	

Skeletal muscles	Vasodilatation	

Peripheral vascular resistance	Increase	

Blood viscosity	Increase	Thrombosis

Platelet aggregation	Increase	Thrombosis

Production and release of nitric oxide	Decrease	Endothelial dysfunction

Total and LDL cholesterol levels	Increase	Accelerated atherosclerosis

Insulin resistance	Increase	Macrovascular complications


Primary effects on cardiovascular physiology include sympathomimetic properties, catecholamine stimulation, and accelerated endothelial dysfunction [97]. Acute use of nicotine causes sympathetic activation and catecholamine release leading to elevations in heart rate, vascular tone, systemic blood pressure, myocardial contractility, and myocardial oxygen demand [98]. Nicotine effects on vascular tone can result in coronary vasoconstriction that can contribute to decreased myocardial oxygen supply and coronary artery spasm [99]. Endothelial dysfunction (impaired function of the cellular lining of arteries leading to arterial narrowing) is one of the major pathogenetic effects of nicotine and results from reduced nitric oxide bioavailability, as well as increased cytokine release and expression of adhesion molecules that promote atherosclerotic plaque development [100]. Nicotine also raises low-density lipoprotein (LDL) cholesterol levels and blood pressure, thereby promoting and accelerating the development of atherosclerosis [101].

Cardiovascular associations with nicotine use in human and animal studies include coronary heart disease, congestive heart failure [102], cardiac dysrhythmias such as atrial fibrillation and ventricular tachycardia/fibrillation [103,104], and thrombosis [105,106].

Tobacco smoking produces a cardiovascular response consistent with nicotine’s effects. The nicotine delivery from ENDS products can be comparable to conventional (tobacco) cigarettes, especially when taking into account the number of puffs, puff volume, duration, and velocity [107,108,109]. Acute physiological effects of ENDS include increases in heart rate, blood pressure, and aortic stiffness [110,111,112]. Further, exposure to ENDS aerosol has been found to have adverse effects on platelet activation and aggregation in both human and animal studies [113], and daily ENDS use is associated with increased risk of myocardial infarction [114].

Although older studies demonstrated no increase in cardiovascular disease among users of snuff, an orally-administered tobacco product, newer studies have found associations between snuff and endothelial dysfunction, decreases in diastolic heart function and an elevated risk of fatal ischemic heart disease and stroke [115,116,117,118,119,120,121].

Further studies on the effects of nicotine on the cardiovascular system are required, as much of what is known about the distinct health effects of nicotine is based on molecular and animal studies.

## Additional Effects of Nicotine

### Acute toxicity

Acute toxicity or poisoning as a result of nicotine exposure is uncommon but does occur [122,123,124]. The toxicity caused by nicotine depends on dose, dose duration and frequency, route of exposure, formulation of the nicotine product, and interpersonal variability. The risk of toxicity has increased with the advent of nicotine-containing solutions used with ENDS products. Nicotine intoxication can lead to various stimulatory symptoms, including tachycardia, hypertension, bronchospasm, salivation, vomiting, diarrhoea, muscular fasciculations, agitation, seizures; and inhibitory symptoms such as bradycardia, arrhythmias, hypotension, muscle paralysis, apnea, and coma. In rare circumstances, nicotine overdose can lead to death [125,126].

### Interference with brain development

From prenatal life to adolescence is a sensitive period for brain plasticity, as well as an important period in terms of regulation of behaviour and cognition. In particular, it is a critical time for initiation of tobacco and nicotine product use, as evidence shows that most chronic smokers started smoking as teenagers or young adults [51,127]. Notably, in the past decade, there has been an alarming increase in ENDS use among adolescents [128,129,130,131].

The effect of nicotine on brain development has been reported in preclinical and clinical studies, including on maternal smokeless tobacco use, which have demonstrated that the nicotinic cholinergic receptors (nAChRs) in the brain play critical maturational roles during adolescence [132,133,134]. Preclinical studies also show that there is an increased number of nAChRs in brain regions that are important for reward, as well as an increase in nicotine-induced dopamine release in limbic regions of the adolescent brain [135,136]. As a result, some behavioural effects of nicotine, such as reward, are more intense in adolescent rats than in adult rats [137,138]. We also know from animal studies that exposure to nicotine during adolescence can lead to long-term changes in brain and behaviour, including increased rewarding effects of other drugs of abuse, decreased level of attention, and mood disturbances [139,140]. These preclinical findings suggest that teen nicotine use may result in similar long-term deleterious effects and are consistent with recent observations of linkages between e-cigarette use and substance use, mental health problems and impulsivity, necessitating further longitudinal assessment of health outcomes in teen and young adult e-cigarette users [141].

### Cognitive and behavioural impairments

Chronic nicotine exposure during adolescence has been associated with cognitive impairments, such as reduced attention span, enhanced impulsivity, and depression [142], lower school grades [143], and school- and substance-related risk behaviours [144]. E-cigarette use initiation has also been associated with lower subsequent academic performance [145].

### Other health effects

In addition to the aforementioned health effects, nicotine exposure has been linked with modified cellular immunity, increased risk of respiratory and gastrointestinal disorders, carcinogenesis and tumour promotion, and negative reproductive and fetal health outcomes such as preterm delivery, stillbirth, and impaired fetal lung development [146].

### Nicotine from second-hand exposure

A number of studies show that exposure to second-hand smoke or ENDS aerosols leads to elevated levels of cotinine in non-smokers [147,148,149,150,151,152], which may indicate absorption of other toxic chemicals and an elevated risk of health harms. The effects of second-hand smoke on cardiovascular health are well-documented, and globally, passive smoking is responsible for nearly 1.2 million deaths every year (4). Studies on passive ENDS aerosol exposure are limited but show that it has the potential to lead to adverse health effects, although less than from exposure to tobacco smoke [151,152,153].

## Nicotine Dependence, Addiction, and Resulting Behavioural Effects

Nicotine is a highly potent addictive compound and the primary addictive chemical component in tobacco products; it is the third most common psychoactive substance used worldwide after caffeine and alcohol [154]. Nicotine dependence is defined, according to WHO’s ICD-11, as ‘*a disorder of regulation of nicotine use arising from repeated or continuous use of nicotine… manifested by impaired ability to control use, increasing priority given to use over other activities and persistence of use despite harm or negative consequences*’ [154]. Addiction can be broadly defined as physical and/or psychological dependency on a drug.

Nicotine in recreational tobacco products leads to sustained use by creating nicotine dependence that makes periods of tobacco/nicotine abstinence (such as during a quit attempt) aversive and uncomfortable [40,155,156]. The risk of developing an addiction to nicotine is determined by various parameters that differ from one nicotine-containing product to another. These include how nicotine is absorbed, the amount of nicotine delivered, the rate at which nicotine is delivered, and the presence of other chemicals and design features that could act synergistically to enhance nicotine’s addictive profile or increase susceptibility to tobacco use [73,157,158].

Commercial tobacco products are manufactured to deliver varying doses of nicotine, causing addiction [159], as most smokers would not use tobacco products if they did not include nicotine [160,161]. The various tobacco and recreational nicotine products in the market, both old and new, have been shown to deliver nicotine in profiles that can lead to addiction, particularly in youth [162,163,164,165,166]. Nicotine addiction due to recreational tobacco and nicotine product use, not only enables continued exposure to other toxic chemicals [167] but has also been associated with alcohol and illicit drug use in youth [168,169,170]. In addition, use of certain products, such as waterpipes and ENDS, has been shown to be a gateway to conventional cigarette smoking uptake [171,172].

### Evidence for nicotine dependence

Continued use of combustible tobacco cigarettes is the most well-studied form of nicotine-self administration, and the evidence of smokers’ impaired ability to control their cigarette use is substantial. The evidence review by the US Surgeon General in 1988 demonstrates that tobacco cigarettes and other forms of tobacco initiate and sustain dependence, nicotine is the drug that causes dependence, and the processes by which nicotine causes dependence are similar to those of heroin or cocaine [173]. There can be no doubt that most people who have smoked cigarettes daily for several months are impaired in their ability to control their tobacco use. For example, in 2018, more than 55.1% of all US adult cigarette smokers had made a quit attempt in the past year, but only 7.5% of those who tried to quit succeeded [174]. Indeed, a recent review of over 130 clinical trials reveals that, even when proven nicotine replacement pharmacotherapies are provided to treatment-seeking cigarette smokers, the overall quit rate is less than 15% [175]. There can be no clearer evidence of ‘impaired ability to control drug use’ than the inability to stop smoking conventional cigarettes despite repeated attempts to do so. There is evidence that other forms of tobacco use that also involve nicotine delivery also result in impaired ability to control use [176,177,178,179].

### Behavioural effects of nicotine dependence

In the brain, nicotine binds to nAChRs leading to the release of dopamine [180,181], a neurotransmitter involved in pleasant feelings. Nicotine also promotes the secretion of norepinephrine, involved in alertness and appetite suppression, and serotonin, involved in mood regulation and transient anti-depressant effects [40,182,183]. Not surprisingly then, for at least some people, first experiences of nicotine may lead to effects that are rated as ‘pleasurable’ [184,185,186]. Indeed, depending upon tobacco use method, these effects may be very pleasurable due to the rapidity with which nicotine is delivered to the brain. For a cigarette, for example, nicotine is delivered to the brain within 10 seconds [187] and this rapid drug delivery may, itself, contribute to drug dependence [188]. Subsequent re-administrations (i.e., smoking again) to maintain these pleasurable effects, perhaps at greater doses due to a diminished effect known as ‘drug tolerance,’ eventually can lead to the uncontrolled use that is the hallmark of dependence.

Another effect of repeated nicotine use is decreased release of neurotransmitters (e.g., dopamine, norepinephrine, serotonin) in the absence of nicotine. This decreased release underlies the characteristic nicotine/tobacco abstinence/withdrawal symptoms that include anxiety, irritability, and depression [189,190]. Aversive abstinence/withdrawal symptoms also contribute to dependence and its hallmark of impaired ability to control use: when an abstaining nicotine user (e.g., a cigarette smoker) experiences these aversive symptoms, they are more motivated to self-administer nicotine (e.g., smoke a cigarette) in order to escape them. Indeed, many smokers report that they smoke to *avoid* these aversive symptoms [191], and this avoidance (or escape, if avoidance is not possible) helps to maintain the nicotine self-administration behaviour [192].

In sum, the hallmark of nicotine dependence is an inability to control nicotine use. This inability to control use is observed most clearly in failed quit attempts but can also be observed in behavioural adaptations that occur when nicotine use is forbidden, such as using nicotine-delivery products like ENDS in an airplane lavatory [193] or selecting entertainment options based on whether or not nicotine use is allowed [194]. The processes that underlie nicotine dependence involve adaptation at the neuronal level and are revealed in the pleasurable effects that are reported when nicotine is first administered (e.g., the first cigarette of the day) and the negative effects that are reported when nicotine is not administered (e.g., during a period of tobacco/nicotine abstinence). Because nicotine dependence is what maintains long-term nicotine tobacco use, it is a key factor in tobacco-caused disability, disease, and death.

## Nicotine Replacement Therapies

Nicotine replacement therapies (NRTs) are first-line medications approved by national health authorities for the management of smoking cessation and contain between 1 to 22 mg across various products [195]. They are also featured in the WHO, as well as many national, Model Lists of Essential Medicines [196]. NRTs are medications designed to deliver nicotine in safe and effective doses, thereby reducing nicotine withdrawal symptoms during tobacco cessation, while avoiding the other toxic chemicals found in all tobacco products and recreational nicotine products [197]. Their use guided by health professionals, NRTs are helpful in managing the symptoms of physical dependence, thereby augmenting the behavioural and cognitive approaches that are also used for smoking cessation. Many smokers require multiple quit attempts before succeeding. While the majority of knowledge about NRT effectiveness is based on cigarette smoking cessation, NRTs can be used for cessation from other forms of tobacco such as smokeless tobacco products [198].

Two types of NRTs are available for managing smoking cessation: short-acting NRTs and the long-acting nicotine skin patch. Short-acting NRTs include the nicotine gum, lozenge, nasal spray, oral inhaler, mouth spray (mist), and sublingual tablets [197,199]. Short-acting NRTs deliver controlled (sometimes self-titrated) doses of nicotine (roughly 0.5 to 2 mg of nicotine are bioavailable per dose through these products) to alleviate the acute cravings for nicotine that occur during a quit attempt. The long-acting nicotine patch, available in three doses, delivers a steady state of nicotine (from 10 to 25 mg per patch) over a period of 16 or 24 hours, providing a more constant relief from abstinence and withdrawal symptoms in smokers [73,200]. The evidence regarding the effectiveness of NRTs in achieving abstinence is well established, and when combined with one another (short-acting and the nicotine patch), with other oral medications such as bupropion, and with cognitive behavioural approaches, NRTs are particularly valuable pharmacological tools for smoking cessation management, particularly given their favourable side effect profile, including for patients with cardiovascular disease [201,202,203,204]. Unlike with ENDS, the long-term safety of NRTs is well documented, and effective protocols to achieve abstinence from both smoking and nicotine have been established [205].

Importantly, NRTs release nicotine in a slow and progressive manner comparatively to tobacco cigarettes and some models of ENDS, resulting in a more gradual absorption into the systemic circulation as well as a lower potential for abuse liability [36,206,207]. In addition, a variety of studies were conducted to understand the ‘abuse potential’ or ‘abuse liability’ of NRTs. Evidence suggests that the use of NRTs outside of therapeutic context is rare [208].

## Impact on Public Health and Product Regulation

### Nicotine as regulated drug versus consumer product

Despite global reductions in tobacco use prevalence, the introduction of ENDS, HTPs, nicotine pouches, and other newer tobacco and nicotine products is impacting tobacco use in youth dramatically in certain parts of the world [69,209,210,211,212]. In addition, despite the lack of strong evidence regarding their efficacy in supporting tobacco cessation, these newer products are being marketed as alternatives to traditional tobacco products with implied benefits for health. Hence, having regulations on not only tobacco products but also their constituents is essential for tobacco control, and this is endorsed widely by the WHO FCTC in its provisions (Articles 9, 10 & 11) [159], with the WHO Framework Convention on Tobacco Control (FCTC) Conference of Parties deciding that Parties should regulate HTPs as tobacco products and regulate new and emerging products by prohibiting or restricting their manufacture, importation, distribution, presentation, sale, and use [213]. Where they are not banned, current regulatory approaches to ENDS vary across the globe (e.g., regulate as tobacco products, imitation tobacco products, medicinal/pharmaceutical products, consumer products, poisons, or as its own separate category).

As per the WHO FCTC, the primary healthcare service for addressing the tobacco epidemic is the cessation and treatment of tobacco dependence [214]. A range of services are available for supporting cessation, including pharmacotherapies such as NRTs that offer safer forms of nicotine delivery that gradually reduce abstinence and withdrawal symptoms and eventually break the addiction to tobacco products. While NRTs are approved by national drug agencies and proven to be effective in cessation, newer products such as ENDS deliver variable and often unregulated amounts of nicotine and have not been approved as cessation aids anywhere in the world.

Evidence suggests that nicotine levels in cigarettes should be limited to a maximum of 0.4 mg/g of tobacco to avoid sustaining nicotine dependence in current smokers and to avoid engendering nicotine dependence in people who initiate smoking [215,216,217]. However, the nicotine present in tobacco products generally ranges from as low as 1.4 mg/g to 20 mg/g. Nicotine content of ENDS liquid ranges from 0.1 to 87 mg/ml [54,218,219]. Moreover, newer generations of ENDS can deliver levels of nicotine that are comparable to those of conventional cigarettes, leading to addiction in non-smokers, or making smokers prone to dual use [220,221,222]. Even e-cigarettes labelled and marketed as electronic non-nicotine delivery systems (ENNDS) contain some amount of nicotine [223].

All tobacco products should be viewed for what they are: nicotine delivery devices that should be regulated through a consistent approach that includes the same level of scientific review and rigor consistent with regulated pharmaceutical drugs, requiring approval by national drug agencies before being allowed to be marketed and sold. Unregulated, insufficiently regulated, or illicit tobacco products, including ENDS, constitute a serious threat to established tobacco control policies, as well as a threat to public and individual health [78]. Hence, regulation is crucial for monitoring and surveillance of production, packaging, labelling, marketing, sale, and use of any product containing nicotine, including approval by national health authorities as a potential tobacco cessation aid before being allowed to be sold.

Importantly, tobacco regulation must be informed by science and refined as newer science becomes available. For example, some jurisdictions regulate ENDS liquid nicotine concentration in an attempt to control nicotine dose inhaled by the user, while ignoring the fact that the nicotine dose delivered to the ENDS user is determined by many other factors (e.g., device power, heating coil dimensions, user puff duration) [224,225]. Thus, regulating only one factor in isolation (i.e., liquid nicotine concentration) can lead users to manipulate other factors (e.g., device power) to defeat the regulatory intent.

A comprehensive nicotine regulation and reduction strategy should be designed to lead to a reduced dependency on tobacco products, regression of tobacco/nicotine addiction among experimenters, and decreased chances of relapse among former tobacco and nicotine users [159]. Hence, regulation of nicotine in tobacco products and for cessation purposes should be consistent to protect and improve public health most effectively.

## Conclusion

Nicotine is a highly addictive substance and a centrepiece of the tobacco pandemic. Unfortunately, the harmful effects of nicotine have been downplayed consistently by the tobacco industry, and promotion of newer products as ‘less harmful’ has expanded nicotine use and addiction, including in youth. While various health harms are due to the many other toxicants found in tobacco smoke, nicotine itself is far from innocuous. A large body of evidence indicates that nicotine, as well as ENDS, are associated with an increased risk of cardiovascular disease and other adverse behavioural and health effects. Data are clear that tobacco and nicotine products are able to initiate and sustain addiction, especially in youth. In fact, most users would not consume the aforementioned products if they did not contain nicotine.

NRTs are a first-line pharmacological intervention for tobacco cessation. They are designed to deliver safe, effective, and controlled doses of nicotine, with minimal side-effects, even for cardiovascular patients, and have low abuse potential. In contrast, evidence suggests that ENDS have a limited effectiveness as smoking cessation aids and have a high abuse potential.

Tobacco and nicotine products are commercialized in various types and forms, many of which remain insufficiently regulated. In particular, manufacturers have sought to circumvent existing tobacco control laws with its newer products and synthetic nicotine.

## Policy Recommendations

Strong and consistent regulations across all products are necessary to protect established tobacco control policies and public health. The World Heart Federation recommends adopting the following measures.

A. For the public, civil society, and health care community:

**Table:** 


MEASURES	OBJECTIVES

All people, particularly those with cardiovascular risk factors or disease, should refrain from using tobacco and non-medical nicotine products.	To prevent first and recurrent cardiovascular events in people living with cardiovascular risk factors or disease. To prevent nicotine addiction and increased risks of health harms.

Educators and community leaders should raise public awareness, particularly through educational activities dedicated to special populations: adolescents, young adults, and women.	To prevent and reduce nicotine addiction. To reduce the number of young patients with cardiovascular diseases. To reduce the number of pregnant smokers. To reduce second-hand exposure to tobacco smoke and ENDS aerosols. To increase community and political support for regulatory measures.

All health care providers should systematically recommend tobacco cessation and provide tobacco cessation services or referrals to patients as standard of care.	To free patients from nicotine addiction and reduce their risks of tobacco-related diseases and their clinical complications. To reduce the tobacco-related socio-economic burden of disease.

The health care community, civil society, and public should constantly call on governments to implement and enforce regulatory measures that protect public health from tobacco and non-medical nicotine products and from the vested interests of the tobacco industry.	To protect and promote the highest standards of health for all people of all ages. To ensure that tobacco regulatory policies are not weakened or influenced by the tobacco industry.


B. For the scientific research community:

**Table:** 


MEASURES	OBJECTIVES

Researchers should study the long-term effects of ENDS, HTP, and other newer recreational nicotine products on cardiovascular health.	To close the knowledge gap on the long-term effects of newer tobacco and recreational nicotine products on cardiovascular health.

Researchers should further probe the drivers of disparities in nicotine and tobacco product use globally and within vulnerable or marginalized communities in a country.	To better understand how to apply tobacco control measures more effectively in order to curb the burden of tobacco uptake and use among more vulnerable populations.

All researchers, academic institutions, and medical and scientific journals should reject tobacco industry collaboration and refrain from publishing and/or presenting studies funded by the tobacco industry.	To prevent tobacco industry interference and promotion of tobacco industry interests. To address conflicts of interest and biased studies. To ensure scientific integrity.


C. For governments:

**Table:** 


MEASURES	RATIONALE

Regulate by prohibiting or restricting the production, distribution, marketing, sale, and use of tobacco and recreational nicotine products.	To improve people’s health by eliminating or reducing their use of tobacco and recreational nicotine products by implementing measures that prevent initiation, promote and support cessation, and discourage consumption of such products.

Require manufacturers of tobacco and nicotine products to provide evidence of safety of their products.	To prevent and reduce health harms and injuries from use of harmful products.

Regulate the amounts of nicotine delivered by tobacco and nicotine products, including delivery devices.	To reduce nicotine concentration and delivery and thereby limit the addiction potential of tobacco and nicotine products and their related adverse health effects. To implement and reinforce Articles 9 and 14 of the WHO FCTC.

Introduce or strengthen pro-health excise taxes for tobacco and non-medical nicotine products.	To increase the prices and reduce the affordability of tobacco and non-medical nicotine products, and thereby discourage and reduce their consumption, especially by youths. To raise additional income for governments and the promotion of health. To implement and reinforce Article 6 of the WHO FCTC.

Require standardized packaging with pictorial health warnings and appropriate labelling on tobacco and nicotine products.	To reduce the appeal of tobacco and nicotine products, especially among youth. To reduce exposure to marketing strategies through packaging. To warn consumers about the dangers of tobacco and nicotine. To implement and reinforce Articles 11, 12, and 13 of the WHO FCTC.

Prohibit the addition of flavouring agents in all tobacco and recreational nicotine products, including ENDS.	To reduce the appeal of tobacco and nicotine products, especially among youth. To prevent the consumption of substances that may be harmful and unsafe for inhalation. To simplify the regulation of e-cigarette solutions. To implement and reinforce Article 9 of the WHO FCTC.

Prohibit the use of aerosol-generating tobacco and nicotine products in indoor public places, workplaces, and public transports.	To protect the public from exposure to second-hand tobacco and nicotine aerosols. To implement and reinforce Article 8 of the WHO FCTC.

Prohibit and monitor all direct and indirect advertising, promotion, and sponsorship of tobacco and recreational nicotine products.	To reduce exposure to marketing strategies that encourage tobacco and recreational nicotine product consumption, including at points of sale and on the internet. To implement and reinforce Articles 5.3 and 13 of the WHO FCTC.

Prohibit and monitor the dissemination of misleading claims on the health effects of tobacco and recreational nicotine products.	To prevent misleading claims that may encourage the consumption of potentially harmful products. To prevent misleading claims on the addictive nature of nicotine and on the effectiveness of newer products as tobacco cessation tools. To implement and reinforce Articles 5.3, 11 and 13 of the WHO FCTC.

Regulate the supply of and retail access to tobacco and nicotine products.	To limit and reduce the availability of tobacco and nicotine products, especially to young people. To reduce exposure to marketing strategies at point of sale.

